# An Unusual Case of Haemophilus parainfluenzae Endocarditis in a Young Patient With Crohn’s Disease

**DOI:** 10.7759/cureus.33770

**Published:** 2023-01-14

**Authors:** Vishrut Shah, Garry Berdichevskiy, Veronica Abello, Hasnan Ijaz, Ihtisham Khalid, Mustafa Rahim, Muntaha Asif, Henry Cusnir

**Affiliations:** 1 Internal Medicine, Westside Regional Medical Center, Plantation, USA; 2 College of Osteopathic Medicine, Nova Southeastern University Dr. Kiran C. Patel College of Osteopathic Medicine, Davie, USA; 3 Internal Medicine, Raleigh General Hospital, Beckley, USA; 4 College of Medicine, Era University, Lucknow, IND; 5 Cardiology, Westside Regional Medical Center, Plantation, USA

**Keywords:** infective endocarditis, anemia, haemophilus parainfluenzae, bacterial translocation, crohn’s disease (cd)

## Abstract

We present a rare case of infective endocarditis (IE) associated with *Haemophilus parainfluenzae* in a 40-year-old male patient with a history of Crohn’s Disease (CD). A complete workup, including an echocardiogram and blood cultures, revealed mitral valve vegetation colonized by *H. parainfluenzae*. The patient was started on appropriate antibiotics with follow-up for outpatient surgery. This case discusses the potential for ectopic colonization of heart valves by *H. parainfluenzae* in patients with CD. The presence of this organism as the offending agent in this patient’s case of IE shines a light on the pathogenesis of CD. Although uncommon, CD-associated bacterial seeding should be a differential when assessing young patients with IE.

## Introduction

Infective endocarditis (IE) is a rare cause of community-acquired and healthcare-associated infections of heart valves [1]. It affects roughly 10,000 to 15,000 patients in the United States every year [1]. It is important to diagnose IE promptly and treat effectively due to its wide variety of predisposing factors and causative microorganisms. Common risk factors for IE include age over sixty, the male sex, intravenous drug use, and compromise of dental health [1]. Additionally, the presence of structural heart disease, indwelling medical devices, previous cardiac intervention, and immune compromise may contribute to the development of IE [1].

Microorganisms that most commonly cause IE include *Staphylococcus aureus*, viridans group streptococci, and enterococci. These microorganisms make up about 80% of all cases [2]. A rarer cause of IE includes *Haemophilus parainfluenzae*, an organism that presents in less than 1% of all cases of IE. It is reported to have a higher predilection for younger patients with the embolic disease or cardiac pathology [3].

We present a case of *H. parainfluenzae* in a young patient with a history of Crohn’s Disease (CD), but no prior history of cardiac pathology or stroke. We believe that *H. parainfluenzae* was able to cause IE in this patient due to the microbiota and cell integrity changes that occur because of CD.

## Case presentation

The patient was a 40-year-old Caucasian male with a past medical history of CD diagnosed at age 19, currently in remission for two years, and not on any medication. The patient’s past medical history also included chronic anemia, COVID-19 infection in December of 2021, partial small bowel resection in 2017, small bowel obstruction status post adhesion removal in 2017, renal cysts, anxiety, and depression. He presented to the hospital accompanied by his mother after being sent to the emergency room by his primary care doctor due to two months of lethargy and low hemoglobin. The patient denied chest pain, palpitations, syncope, and abnormal bleeding. Additionally, he denied any recent dental work with gingival alteration, skin infection, or recent gastrointestinal procedures.

Blood examinations, as seen in Table 1, showed an abnormal hemoglobin (3.9 g/dL), a low hematocrit (13.2%), and a normal white blood cell count (8,200 mg/dL). Blood chemistries revealed abnormal blood urea nitrogen (72 mg/dL), creatinine (3.09 mg/dL), and glomerular filtration rate (23 mL/min/1.73 m^2^). In addition, there were elevations in D-dimer (4086 ng/mL) and brain natriuretic peptide (4300 pg/mL) levels, and troponin was positive at 0.145 ng/mL. A fecal occult blood test was positive, but the patient denied any melena or bloody stools.

**Table 1:** Laboratory Tests Done During Patient’s Hospital Stay

Laboratory Study	Result	Reference Range
Hemoglobin	3.9	12-15.5 g/dL
Hematocrit	13.2	14-18%
White blood cell count	8,200	4,500-11,000 mg/dL
Platelet count	186,000	150,000-450,000/µL
Sodium	133	135-145 mmol/L
Potassium	3.9	3.5-5 mmol/L
Calcium	7.5	8.6-10.2 mg/dL
Phosphorus	5.8	3-4.5 mg/dL
Blood urea nitrogen	72	8-21 mg/dL
Creatinine	3.09	0.7-1.2 mg/dL
Glomerular filtration rate	23	100-130 mL/min/1.73 m^2^
Glucose	129	<140 mg/dL
Albumin	2.5	3.4-5.4 g/dL
Aspartate aminotransferase	30	10-40 units/L
Alanine aminotransferase	37	7-56 units/L
D-dimer	4086	<500 ng/mL
Brain natriuretic peptide	4300	<125 pg/mL
Troponin	0.145	<0.04 ng/mL
Fecal occult blood test	Positive	Negative

A chest X-ray showed no evidence of acute infiltrates, pulmonary edema, or pleural effusions. A physical exam revealed bilateral lower extremity edema. However, bilateral lower extremity venous Doppler was negative for deep vein thrombosis. The patient further stated that his last CD flare-up was two years ago. His last colonoscopy was also two years ago, and showed mild redness secondary to CD, but was negative for polyps. The patient was admitted to the hospital for further management and evaluation.

**Figure 1:**
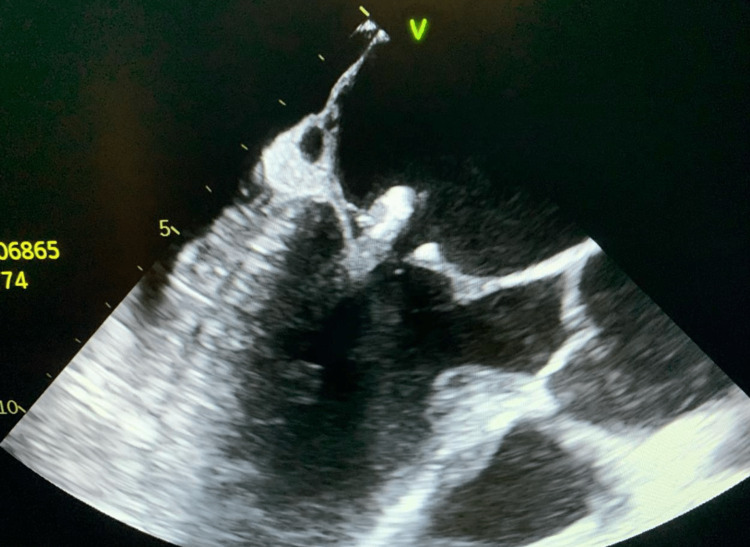
Transesophageal Echocardiogram Performed Demonstrating a Posterior Mitral Valve Leaflet Vegetation

The patient was diagnosed with severe symptomatic anemia secondary to a suspected gastrointestinal bleed, for which he received six units of packed red blood cells throughout his stay. Gastroenterology was consulted to evaluate for active bleeding. They recommended supportive care, and an outpatient endoscopy and colonoscopy once stable with a negative cardiac workup. Cardiology was consulted, and an electrocardiogram was done, which revealed a non-ST segment elevation myocardial infarction likely due to ischemia from symptomatic anemia. A review of past admissions revealed positive blood cultures for *H. parainfluenzae* one month prior to admission. A transthoracic echocardiogram (TTE) was performed to evaluate for new vegetation secondary to bacteremia. However, the TTE was unremarkable. Therefore, a transesophageal echocardiogram (TEE), as seen in Figure 1, was conducted, which demonstrated a posterior mitral valve leaflet vegetation 1.3 cm in size. Additionally, the TEE showed that the current vegetation was superimposed on a calcified mitral valve, which suggested a reinfected valve. The patient was initiated on empiric intravenous vancomycin pending blood cultures.

Infectious disease was consulted regarding concern for IE. Blood cultures were performed using chocolate agar media, and were positive for *H. parainfluenzae*. Thus, empiric vancomycin was discontinued, and intravenous ceftriaxone was initiated. Cardiothoracic surgery was consulted for valve repair, and it was determined that surgery would be planned in an outpatient setting once the patient was discharged.

## Discussion

Most patients with IE present with a fever. However, malaise, headache, myalgias, arthralgias, night sweats, abdominal pain, and dyspnea are also common [4]. Cardiac murmurs may also be present, and depend on the location of the infected valve. IE may develop because of bacteremia or cardiac injury. Such insults can serve as a nidus of infection for microorganisms to grow and form vegetation. Prolonged microbial growth activates coagulative pathways that lead to fibronectin deposition. This can further exacerbate the growth of infective microorganisms [5]. The most common pathogens involved in IE are staphylococcus, streptococcus, and enterococcus [1]. Other culprits include fungi and a group of fastidious gram-negative bacilli collectively referred to as Haemophilus species, Aggregatibacter species, *Cardiobacterium hominis, Eikenella corrodens, *and Kingella species(HACEK) organisms, which make up approximately 2% of reported cases [6].

A combination of clinical presentation, microbiology, and imaging is used to diagnose IE [4]. The Modified Duke Criteria is divided into major and minor criteria. Major criteria include positive blood cultures and evidence of IE on imaging. Minor criteria include IE risk factors, fever, vascular and immunological phenomena such as Janeway lesions, Roth spots, Osler nodes, and microbiological evidence of infection [2]. A diagnosis is made when a patient presents with two major, one major and three minor, or five minor criteria of IE [2]. Treatment of IE involves targeted antibiotic therapy and a multidisciplinary evaluation for surgery [7].

Genomic analysis of gastrointestinal microbiota revealed that *H. parainfluenzae* is part of a group of organisms that increase in number in patients with CD [8]. It is suggested that this could be in part due to environmental changes caused by the disease, allowing the organisms increased chances of survival [9]. What is interesting, however, is that most of these organisms are predominantly part of the human oral microbiota. *H. parainfluenzae* accounts for 74% of pharyngeal Haemophilus species in healthy adults and children [10].

While the exact role of normal microbiota in CD is still an immensely researched topic, it is suggested that the physical migration of the oral microbiota is in part responsible for its increased presence in the gut in patients with CD [11]. Additionally, these microorganisms develop a propensity to colonize the inflamed portions of intestinal mucosa associated with CD. This was shown in a genomic sequencing study where inflammatory bowel disease samples cleared of fecal material revealed persistently elevated numbers of translocated microorganisms [12]. This suggests that in our patient, *H. parainfluenzae* may have been able to migrate from the oral cavity, and then colonize and grow in areas of intestinal inflammation secondary to his CD.

Loss of cellular integrity of the gut epithelium is common with injury and allows ions, metabolites, and bacteria to enter the bloodstream from the bowel [13]. This is particularly true in patients with CD, and it has been shown that gram-negative bacteria, such as *H. parainfluenzae*, can use lipopolysaccharides to cause further inflammation and invade the gut endothelium to cause bacteremia [14]. Once inside the blood, the microorganism can travel to cardiac valves and develop into IE. Therefore, we propose that it was CD-associated changes in intestinal inflammation, and ectopic *H. parainfluenzae* translocation to the gut, that predisposed our patient to develop bacteremia that led to his IE.

## Conclusions

*H. parainfluenzae* is a rare cause of IE, more common in younger patients with cardiac and embolic pathology. However, in the absence of these pathologies, it is important to consider other aspects of a patient's medical history. This report demonstrates there may be a risk of IE in patients with CD, and the mechanism by which *H. parainfluenzae* can enter the bloodstream because of the chronic inflammation.
